# Accelerating the transition of new tuberculosis drug combinations from Phase II to Phase III trials: New technologies and innovative designs

**DOI:** 10.1371/journal.pmed.1002851

**Published:** 2019-07-09

**Authors:** Geraint Davies, Martin Boeree, Dave Hermann, Michael Hoelscher

**Affiliations:** 1 University of Liverpool, Liverpool, United Kingdom; 2 Radboud University, Nijmegen, the Netherlands; 3 The Bill and Melinda Gates Foundation, Seattle, Washington, United States of America; 4 Division of Infectious Diseases and Tropical Medicine, Klinikum of the University of Munich, Munich, Germany; 5 German Center for Infection Research (DZIF), partner site, Munich, Germany

## Abstract

Geraint Davies and colleagues discuss the potential for innovative early-phase clinical trial methods and technologies to reduce risk and speed up drug development for tuberculosis.

**Summary points**

- Preclinical models of tuberculosis have significant limitations in selecting composition and duration of regimens for tuberculosis.
- Innovative early-phase clinical trial methodologies and technologies have the potential to reduce risk and accelerate drug development in tuberculosis.
- Phase IIA monotherapy studies are optional for proof of concept but may be useful for dose-finding in conjunction with pharmacokinetic–pharmacodynamic methods.
- Innovative Phase IIB designs are increasingly common in tuberculosis drug development, utilising multiarm selection designs, sometimes in an adaptive format.
- Novel biomarkers including liquid culture and nucleic acid amplification appear capable of replacing conventional solid culture in early-phase development.
- Phase IIC and ultrashort noninferiority designs attempt to mitigate the problem of estimating duration of treatment regimens from Phase II results alone.

## Background

**Fig 1:**
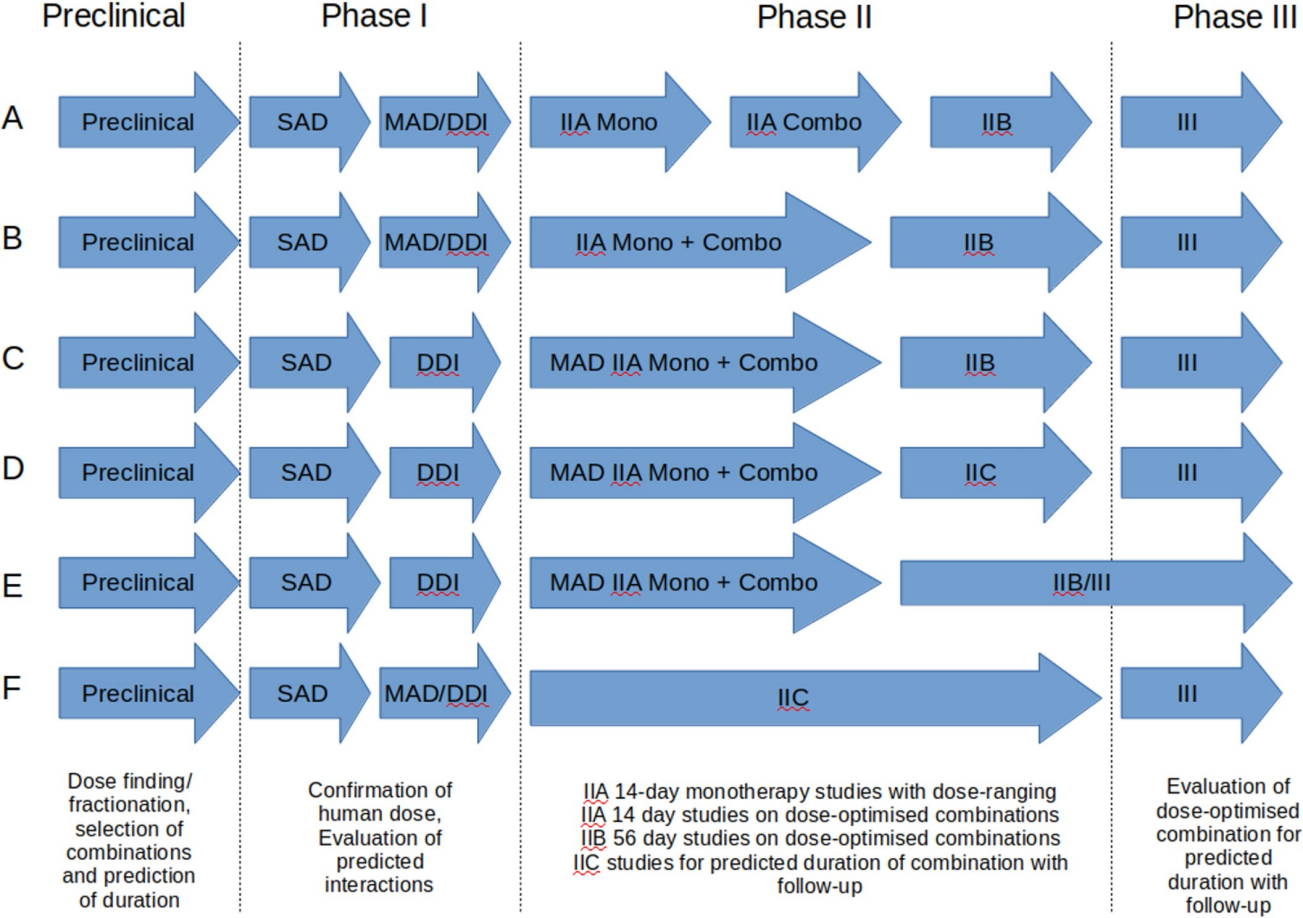
Elements of critical pathways for clinical development of TB drugs illustrating selected alternatives: (A) Current standard approach, (B) 14+14 IIA design, (C) Dose-ranging 14+14 IIA design incorporating MAD PK in patients, (D) Phase IIC design, (E) Seamless IIB/III design, and (F) IIC design with no monotherapy (‘Mono’) stage. Combo, combination therapy; DDI, drug–drug interaction study; MAD, multiple ascending dose; PK, pharmacokinetic; SAD, single ascending dose; TB, tuberculosis.

Tuberculosis (TB) remains the single biggest killer among infectious diseases, and treatment is prolonged, complex, and vulnerable to the development of resistance. Innovation in TB therapy is desperately needed, but the transition from first-in-patient studies to pivotal clinical trials in the treatment of TB is plagued by uncertainty for several reasons. In preclinical development, a limited understanding of mycobacterial physiology in vivo and an inability to closely mimic human pathology in animal models limit confidence in the translational predictions that may be used to plan early-phase trials. Furthermore, these trials face problems in accurately and rapidly measuring response to treatment in accessible clinical samples because of a continuing reliance on mycobacterial culture of sputum and constraints posed by emergence of resistance during prolonged monotherapy. Irreversibility of the response precludes the use of crossover designs, and the need for combination treatment regimens containing three or more drugs limits the scope of dose-finding studies in clinical development. Anti-TB drugs have widely differing and sometimes poorly understood mechanisms of action capable of producing qualitatively different patterns of response, as well as variable physicochemical characteristics, distribution, and toxicity profiles. It is important, therefore, to recognise that a strategic approach to development pathways for different drugs may need to reflect these differences while identifying and preserving the core elements of a successful critical path to registration. At the same time, it is generally accepted that drug developers in TB need to focus more on codevelopment of regimens rather than drawing only on the lessons learned from development of individual drugs, which poses additional challenges in evaluation of both safety and efficacy [1]. Policymakers are increasingly seeking to integrate innovative clinical trial approaches into their decision-making to facilitate early and effective deployment of the best regimens [2], as emphasised by this collection. In the TB context, the key information to be gained in Phase II development includes obtaining initial proof of concept, finding the optimal dose for individual agents, selecting the best combinations of drugs to be further tested in Phase III trials, and predicting the likely necessary duration of a future regimen. Regulators have recently demonstrated openness to innovative approaches in these areas and flexibility around when and how key information on efficacy and safety of individual agents may be obtained during development. A number of new directions promise to improve the means by which these goals are currently achieved in TB trials, which we present in this paper (Fig 1).

## Phase IIA monotherapy and combination studies

The traditional approach to Phase IIA (‘first-in-patient’) studies in TB since the 1980s has been short-term trials of treatment based on reduction of colony-forming units of *M*. *tuberculosis* in repeated sputum samples over the first 14 days of treatment, termed ‘early bactericidal activity’ (EBA) studies [3]. Such studies, using small sample sizes (10–15 per arm), have been used for initial dose-finding in humans, studying pharmacodynamic interactions between drugs, and more recently for selecting combinations, often based on indications from various mouse models [4]. They are economical relative to other phases of development and have the potential to demonstrate the contribution of individual drugs in humans prior to commencing study of combinations. This proof of concept has been particularly important in TB, given that pharmacodynamics cannot be investigated in healthy volunteer studies. However, a number of drugs that are known to influence long-term outcomes of treatment have little or no impact on quantitative bacteriology in the early phase of therapy [5,6], suggesting that positive results in a Phase IIA trial may be dispensable for some important agents, though it remains uncertain whether these agents can currently be clearly identified in advance during preclinical development. In addition, although a small Phase IIA trial may contribute to initial evaluation of safety in patients under conditions of monotherapy, their small sample size contributes only modestly to expanding the safety database from Phase I studies.

For these reasons, the value of Phase IIA monotherapy studies has been questioned, since positive results are neither sufficient nor even necessary for progression in development. They do represent, however, the first and last time that evidence on the contribution of individual drugs can be obtained during a development programme, and for this reason, a number of modifications to improve this approach have been suggested. Since it is clear that Phase IIA studies are prone to misinterpretation due to differing patterns of pharmacodynamics and relatively high interindividual variability, modern studies are usually conducted for a full 14 days [7], which is widely considered to be the ethical limit for monotherapy, beyond which the risk of generating resistance is too great. This period appears long enough to capture the full pharmacodynamic behaviour of drugs in the early phase of treatment. A major innovation in recent years has been the systematic application of pharmacokinetic–pharmacodynamic (PK-PD) methods to the analysis of Phase IIA studies, which have helped to discriminate different patterns of response, account for important baseline prognostic variables, and improve the power of comparisons while also clarifying dose-response relationships using pharmacokinetic data.

Another innovation is the replacement of traditional bacteriology performed on selective solid media by other methods, such as liquid culture in the mycobacterial growth indicator tube (MGIT) system, which appears to have similar variability to solid culture and may remain positive for longer, providing a more plausible link to later-phase studies [8]. However, the relationship between colony counts on solid media and time to positivity in liquid culture changes over time, and the two measurements may not be completely interchangeable in longer studies [9]. Most recently, molecular assays promoted as candidates to replace culture-based techniques have begun to be evaluated. The DNA-based Xpert MTB RIF assay appears to lack the dynamic range required to be useful in early-phase studies, though it has been used in dose-response analysis in one study [10,11]. An assay based on 16S rRNA has, however, been shown to correlate well with quantitative cultures and persists longer during treatment [12,13], whereas full profiling of the transcriptome of *M*. *tuberculosis* in sputum may also enable important qualitative changes in bacterial physiology to be captured [14,15]. A novel assay for quantifying lipoarabinomannan in sputum has also recently been developed, though not as yet fully evaluated. Finally, imaging endpoints based on positron emission tomography–computed tomography (PET-CT) have been suggested as a complement or replacement for bacteriological measurements [16], though data on the value of this approach are so far limited, and the required technology is not widely available where TB is common.

An important recent trend has been for Phase IIA studies to go beyond 14-day proof-of-concept and dose-finding studies for individual drugs, and increasingly, focus has shifted to evaluating combinations of drugs. This is important because a recent meta-analysis of Phase II studies in TB noted that there was almost no overlap between the regimens studied in Phase IIA and Phase IIB [17], suggesting that decisions on drug selection and combination have historically been based largely on considerations other than performance in EBA studies, usually combination studies in the mouse model. However, uncertainty remains about how predictive such studies may be in humans, and the ability to confirm and select among at least a subset of promising regimens in clinical studies would clearly be desirable. Combination Phase IIA studies may follow separate initial monotherapy studies [18], or a monotherapy run-in of 14 days may be followed by a 14-day study of combinations (14+14 design) [19]. Such an approach representing a fusion of dose-finding and selection of combinations appears to be time-efficient and would represent a more significant expansion of the safety database in terms of numbers and duration. A similar 7+7 design has been successfully employed in a formal maximum tolerated dose study for dose-finding of rifampicin [20]. However, although the duration of exposure of patients to novel or higher doses of existing agents must clearly be determined by preexisting preclinical toxicology data, given that ethical concerns about resistance are minimised by the use of combinations, restricting Phase IIA combination studies to a period of 14 days appears arbitrary, restricting information about longer-term response. It also remains unclear whether some of these studies are large enough to formally discriminate among regimens reliably.

## Phase IIB studies

Traditionally, Phase IIB studies in TB have relied on the endpoint of culture conversion at 8 weeks, based on its simplicity and the abundance of historical data showing that it is a useful, yet imperfect, surrogate endpoint for long-term treatment response [21,22]. However, reliance on this binary endpoint typically mandated relatively large sample sizes for such trials, for statistical reasons, and posed problems in using them for dose-finding and selection of combinations [23,24]. It also resulted in an inability to directly compare quantitative bacteriological results obtained in Phase IIA studies with the 8-week endpoint, meaning that there was almost no direct translational linkage between the two.

**Fig 2:**
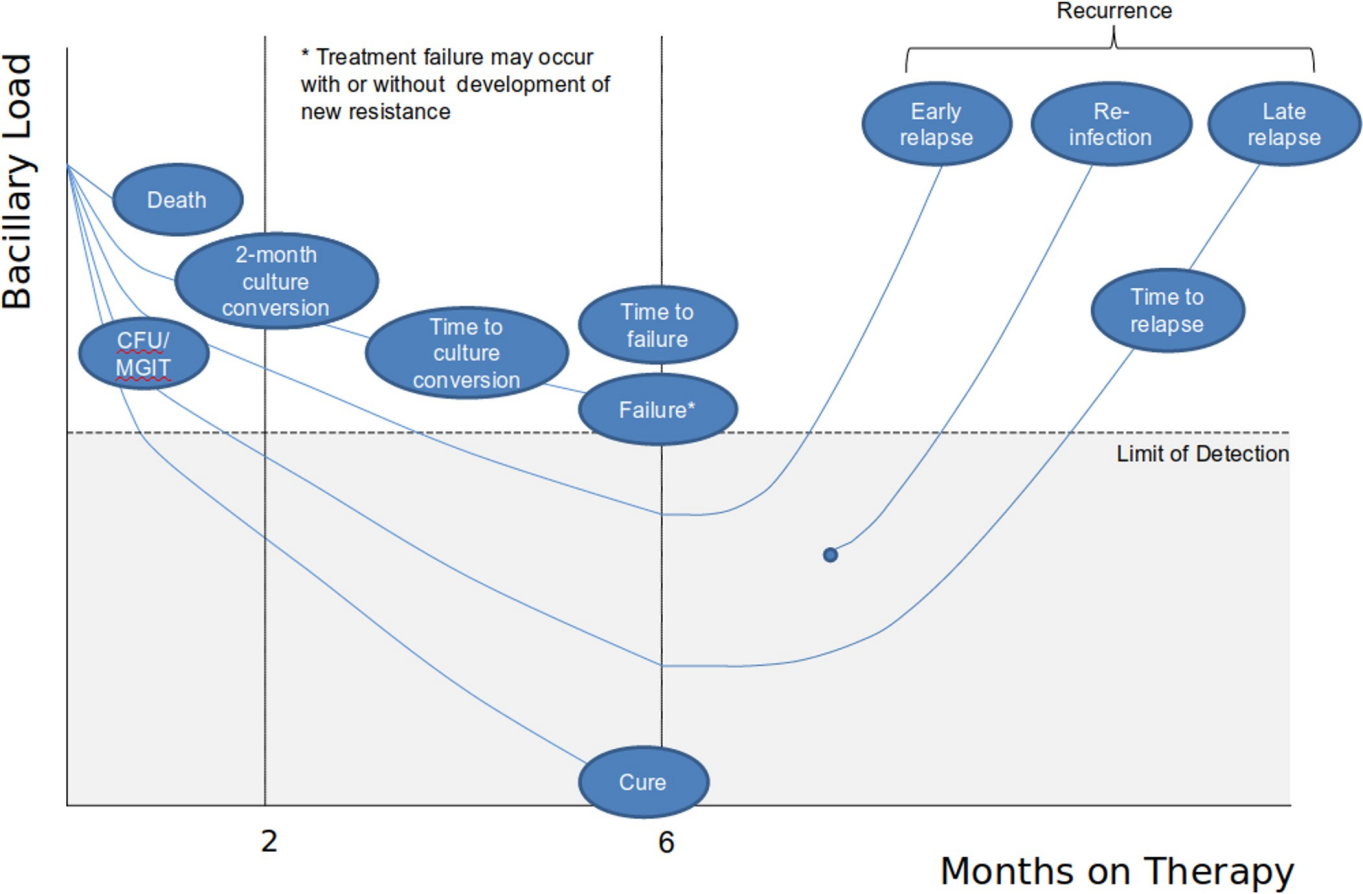
Schematic illustration of alternative bacteriological approaches to measurement of elimination of organisms in respiratory specimens over time in clinical trials of TB. After conversion of cultures to negative in the first weeks of treatment, subsequent stable cure is defined only by the absence of relapse (return of positive cultures). CFU/MGIT, modelling of colony-forming units or mycobacterial growth indicator tube data; TB, tuberculosis.

Over the last decade, however, new approaches to Phase IIB studies based on longitudinal statistical modelling of quantitative bacteriology, time to positivity in MGIT, or time-to-culture conversion data have been increasingly adopted by investigators (Fig 2) [25–27]. These studies have continued to be performed over the first 8 weeks of treatment but have employed more intensive sampling of sputum at earlier time points. As experience with effect sizes using these novel analyses has grown, such approaches have facilitated greater flexibility and economy in trial designs with reduced sample sizes (40 per arm) [28], although as yet they lack the support from long-term studies that is enjoyed by 8-week culture conversion. However, because these outcomes are measured on a continuous rather than binary scale, they offer longer-term advantages in terms of validation over the 8-week endpoint, which is approaching 100% culture conversion in the comparator arm as regimens improve in efficacy. The confidence attached to the 8-week endpoint is not easily transferred to other single time points with which there is less experience. Worthy of special emphasis is the importance of laboratory quality. For any of these dichotomous and quantitative bacteriologic endpoints to be maximally informative, significant investment in and oversight of quality of microbiologic assays and laboratory procedures are essential.

The advent of these more quantitative endpoints has led to reconsideration of the purpose and design of Phase IIB trials and their possible use to achieve some of the objectives of Phase IIA trials of combination regimens. A multiarm Phase IIB trial method has been successfully used to select among members of the fluoroquinolone class substituted into the first-line combination regimen for progression to Phase III [29]. The results of these pivotal trials initially appeared disappointing and highlighted difficulties in accurately predicting duration of regimens from effect sizes in these novel IIB designs. However, recent pooled reanalyses are suggestive that the treatment-shortening potential of these regimens may in fact be confined to a majority subgroup of patients [30]. Similar Phase IIB designs have subsequently been used to select regimens containing the novel agents pretomanid and bedaquiline for Phase III trials, though the latter have not yet been completed [31]. More recently, three other similar Phase IIB trials were used for dose-finding among the rifamycins, rifampicin, and rifapentine [32–34]. All these trials were explicitly designed and analysed using a PK-PD modelling approach, and two of them successfully demonstrated relationships of bacteriological response with dose and/or plasma concentrations, which have also been used to plan ongoing Phase III trials.

The demonstration of the feasibility of Phase IIB designs for dose-finding and selection of combinations has naturally led to exploration of adaptive designs that may offer the opportunity to test a broader range of both combinations and dose levels without increasing the number of patients enrolled prohibitively. An early attempt to apply this approach was unsuccessful and pointed to limitations on opportunities to adapt when using an 8-week primary endpoint [35]. However, a multiarm multistage (MAMS) design, similar to those developed in oncology, has been subsequently successfully implemented, eliminating two of its five arms, which contained the investigational agent SQ-109, prior to full enrollment [36,37]. This study demonstrated that meaningful adaptation in TB trials is possible but also drew attention to some of the challenges, particularly delays in receiving culture information, which must be balanced against a relatively slow rate of recruitment and the challenges of medication management of many diverse treatment regimens, which would usually preclude complete masking of treatment for purposes of safety assessment.

## Predicting long-term outcome and duration

In order for regimens to be reliably selected in Phase II, investigators need to have reasonable confidence that the intermediate bacteriological endpoints on which they currently rely can be trusted to correctly predict treatment effects on definitive long-term outcomes, such as treatment failure and relapse. Several analyses have addressed this question, largely focusing on the 8-week culture conversion endpoint. In an early meta-regression analysis of the historical British Medical Research Council trials, the 8-week endpoint was found to be a reasonable predictor of long-term outcome for rifampicin-based regimens [20]. An extension of this analysis confirmed these results and developed a prediction model for the duration of a regimen required to produce acceptable rates of relapse, which appeared to perform well when applied to new datasets involving classes of drugs not included in the training set [38]. Finally, an analysis comprising all historical regimens included in TB trials confirmed the usefulness of this meta-regression approach while showing that the relevant relationships may be different for regimens that do or do not contain rifampicin [39]. These data, while supporting the utility of a meta-regression approach, suggest that 8-week culture conversion is a useful but imperfect surrogate endpoint for long-term treatment response and may be subject to drug class effects. Although this is also likely to be true for other intermediate bacteriological endpoints, data to support a similar analysis based on time-to-event or bacillary elimination rates are as yet too sparse to replicate this approach.

Alternatives to this indirect approach involve collecting varying degrees of follow-up data after Phase IIB trials have been completed. The simplest way to do this is to follow all the patients enrolled in a Phase IIB trial to the end of their complete regimen and for a defined period post-treatment, usually 12 months. This design, termed STEP Phase IIC by its proponents, permits estimation of a Bayesian prediction interval for the likely results of a future Phase III trial, with the advantage that the prediction is less dependent on intermediate outcomes than in the meta-regression approach [40]. However, slightly larger sample sizes (80 per arm) than those typically used for a Phase IIB selection design are desirable, and the intermediate results are still used as a threshold to prevent participants being exposed to very poorly performing regimens. The Phase IIC design is thus related to that used in the Phase III TRUNCATE-TB trial, which is evaluating ultrashort 2- to 3-month regimens in a similar way but with fully powered noninferiority comparisons for which a much larger sample size is required [41]. A third approach, which has not yet been implemented in TB, is a fully seamless Phase II/III design in which adaptive evaluation of regimens in the Phase II stage is followed by enrichment of the successful arms with additional participants in the Phase III stage to achieve appropriate power for comparisons on the long-term outcomes in the selected arms [42].

Preclinical information could also be a useful source of data for initial predictions of duration of regimens, based either on bacterial elimination rates or relapse experiments. An approach based on translational PK-PD modelling of mouse data has been used to predict the results of a number of clinical trials with reasonable success [43]. Similar approaches combining preclinical and clinical data using Bayesian approaches have the potential to guide early-phase clinical development decisions in real time.

## Safety considerations

Similarly to other complex therapeutic areas, effective therapy for TB relies on combining at least three and as many as seven drugs. Although the safety profile of many existing or repurposed drugs is relatively well characterised, the concept of universal regimens combining multiple novel agents raises some issues of interpretation of toxicity signals, which may only be clearly resolved when there are relatively extensive safety data generated during Phase I and II monotherapy studies. However, although such studies can address short-term toxicities, longer-term issues can only be addressed by good laboratory practice (GLP) standard toxicity studies in nonhuman animal models conducted at the appropriate time in the development programme. Regulatory guidance recognises this problem and specifies that, provided that complete preclinical development is also carried out for each component of a combination, an animal combination toxicity study equivalent to the duration of planned clinical trials, up to a maximum of 90 days, in a single relevant species would be sufficient to support marketing [44]. In some cases this requirement may be waived if no overlapping toxicities are observed in the preclinical programmes for each component, but further studies may be necessary if unexpected toxicities not observed with any of the components occur in the combination study.

## Conclusions

Methods for transitioning of TB drugs and regimens through Phase II to Phase III have evolved rapidly in the last decade. Innovation in clinical trials methodology and evaluation of biomarkers have increased the confidence with which multiarm selection and adaptive designs have been adopted by the TB trials community. Although clinical monotherapy studies do not appear mandatory from a regulatory point of view, they may assist developers when significant uncertainty in preclinical development requires strong proof of concept in humans. The recently increased emphasis on development of combinations, however, may benefit from an increased variety of possible trial designs based on longitudinal bacteriological responses over various durations of therapy and making use of adaptation within sustainable clinical trial platforms. Acknowledging the imperfect nature of current intermediate outcomes, investigators are also seeking to increase confidence in Phase III planning by obtaining limited long-term follow-up in extended Phase IIC designs or in bypassing Phase IIB altogether. Translational modelling approaches may complement these data by explicitly bringing preclinical data to bear on clinical development decisions. These innovations promise to reduce risk and accelerate early-phase clinical development in TB, increasing the confidence that regimens selected for Phase III trials contain the right drugs at the right doses and maximising the possibility of successfully achieving reductions in treatment duration for patients.

## Abbreviations

Combo: combination therapy
DDI: drug–drug interaction study
EBA: early bactericidal activity
GLP: good laboratory practice
MAD: multiple ascending dose
MAMS: multiarm multistage
MGIT: mycobacterial growth indicator tube
PET-CT: positron emission tomography–computed tomography
PK-PD: pharmacokinetic–pharmacodynamics
SAD: single ascending dose
TB: tuberculosis
